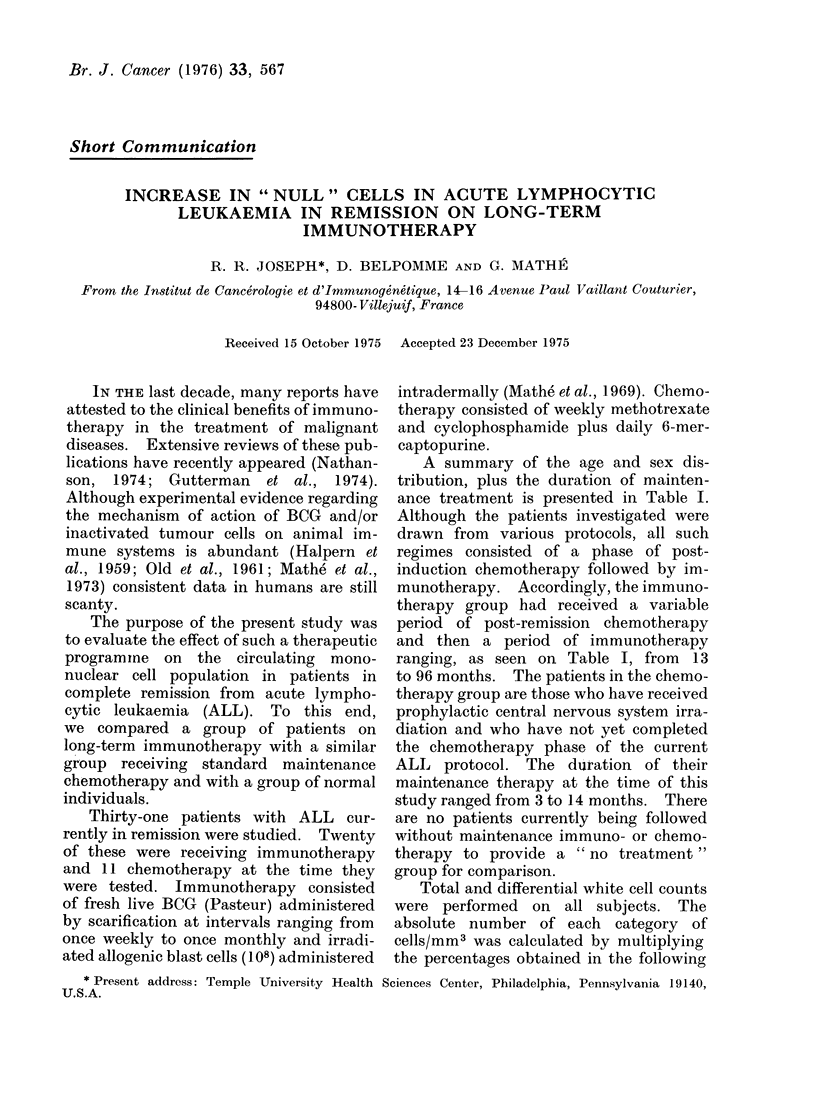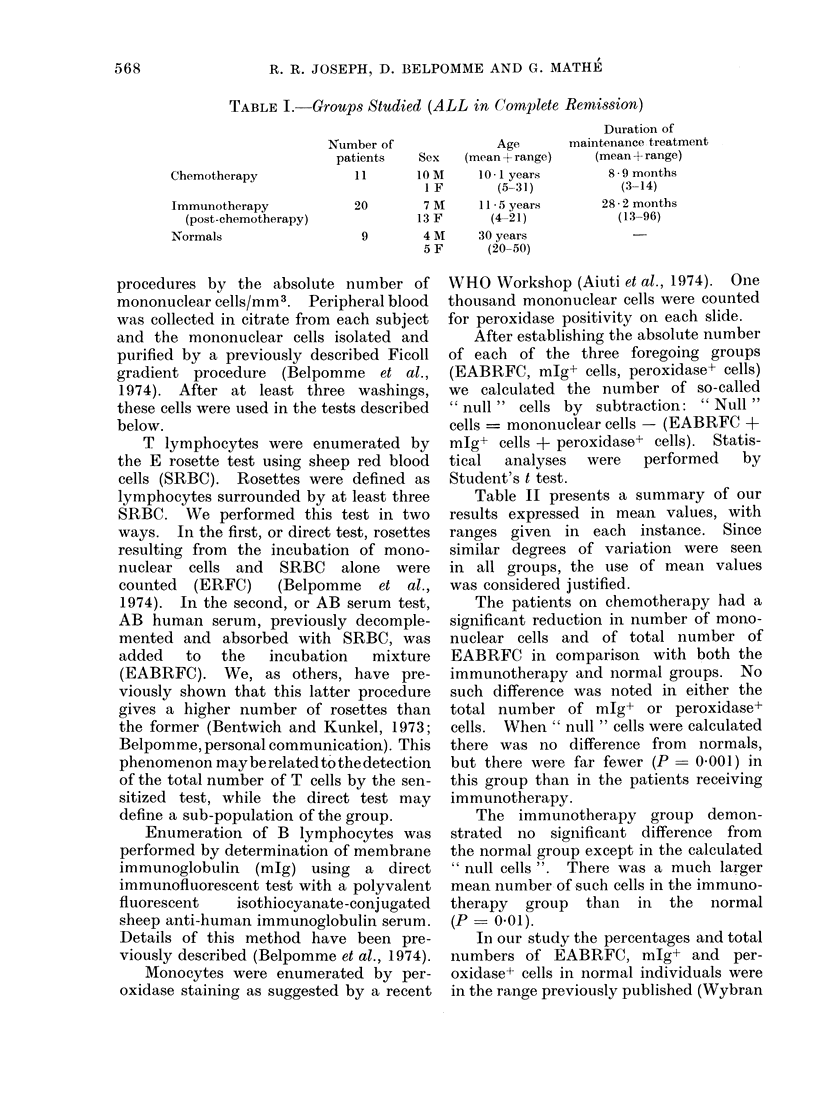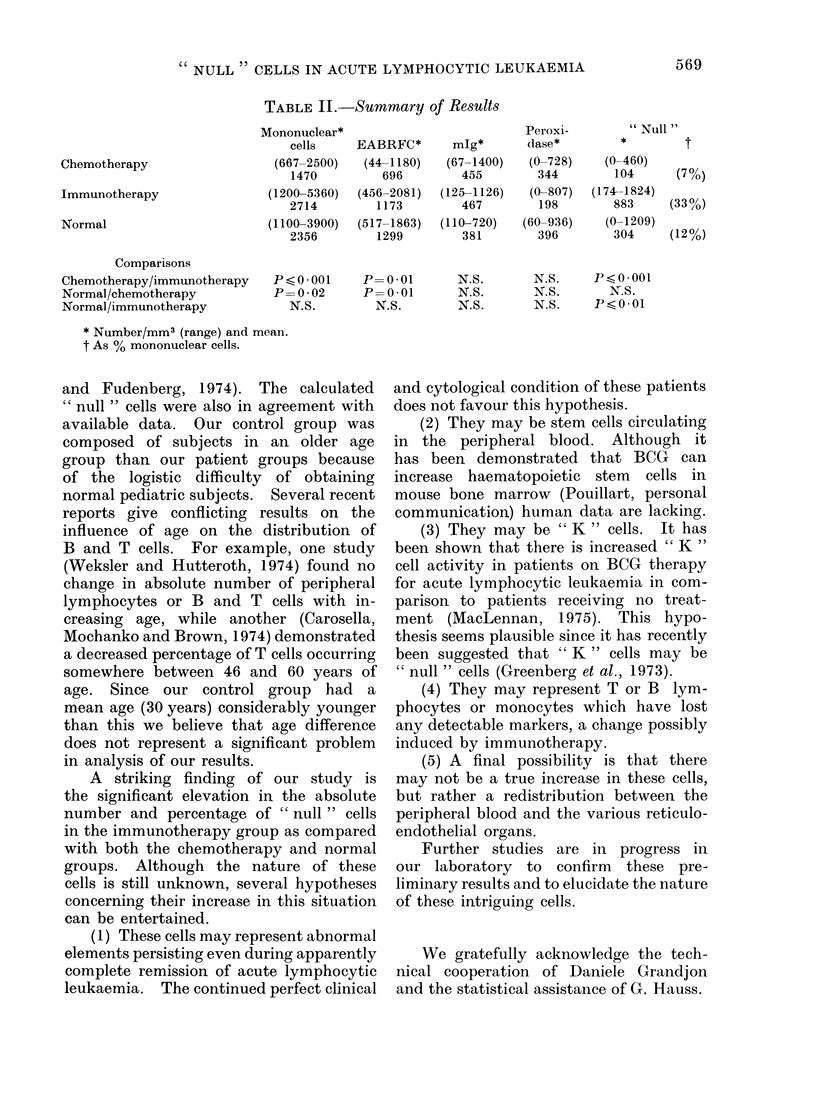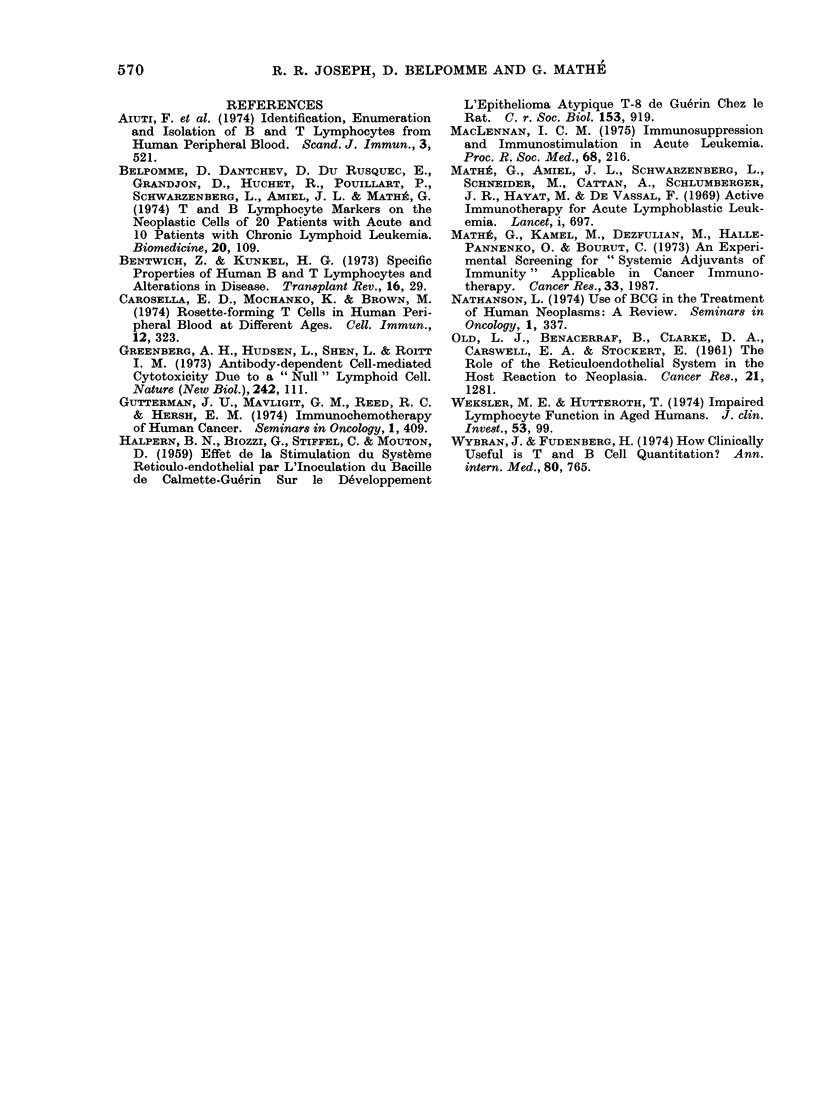# Increase in "Null" cells in acute lymphocytic leukaemia in remission on long-term immunotherapy.

**DOI:** 10.1038/bjc.1976.91

**Published:** 1976-05

**Authors:** R. R. Joseph, D. Belpomme, G. Mathé


*Br. J. Cancer* (1976) **33**, 567

***Short Communication***

### INCREASE IN "NULL" CELLS IN ACUTE LYMPHOCYTIC LEUKAEMIA IN REMISSION ON LONG-TERM IMMUNOTHERAPY

R. R. JOSEPH*, D. BELPOMME AND G. MATHÉ

*From the Institut de Cancérologie et d'Immunogénétique,* 14–16 *Avenue Paul Vaillant Couturier,* 94800-*Villejuif, France*

Received 15 October 1975 Accepted 23 December 1975

IN THE last decade, many reports have attested to the clinical benefits of immunotherapy in the treatment of malignant diseases. Extensive reviews of these publications have recently appeared (Nathanson, 1974; Gutterman *et al.*, 1974). Although experimental evidence regarding the mechanism of action of BCG and/or inactivated tumour cells on animal immune systems is abundant (Halpern *et al.*, 1959; Old *et al.*, 1961; Mathé *et al.*, 1973) consistent data in humans are still scanty.

The purpose of the present study was to evaluate the effect of such a therapeutic programme on the circulating mononuclear cell population in patients in complete remission from acute lymphocytic leukaemia (ALL). To this end, we compared a group of patients on long-term immunotherapy with a similar group receiving standard maintenance chemotherapy and with a group of normal individuals.

Thirty-one patients with ALL currently in remission were studied. Twenty of these were receiving immunotherapy and 11 chemotherapy at the time they were tested. Immunotherapy consisted of fresh live BCG (Pasteur) administered by scarification at intervals ranging from once weekly to once monthly and irradiated allogenic blast cells ($10^8$) administered intradermally (Mathé *et al.*, 1969). Chemotherapy consisted of weekly methotrexate and cyclophosphamide plus daily 6-mercaptopurine.

A summary of the age and sex distribution, plus the duration of maintenance treatment is presented in Table I. Although the patients investigated were drawn from various protocols, all such regimes consisted of a phase of post-induction chemotherapy followed by immunotherapy. Accordingly, the immunotherapy group had received a variable period of post-remission chemotherapy and then a period of immunotherapy ranging, as seen on Table I, from 13 to 96 months. The patients in the chemotherapy group are those who have received prophylactic central nervous system irradiation and who have not yet completed the chemotherapy phase of the current ALL protocol. The duration of their maintenance therapy at the time of this study ranged from 3 to 14 months. There are no patients currently being followed without maintenance immuno- or chemotherapy to provide a "no treatment" group for comparison.

Total and differential white cell counts were performed on all subjects. The absolute number of each category of cells/$mm^3$ was calculated by multiplying the percentages obtained in the following

* Present address: Temple University Health Sciences Center, Philadelphia, Pennsylvania 19140, U.S.A.

TABLE I.—*Groups Studied (ALL in Complete Remission)*

| | Number of patients | Sex | Age (mean + range) | Duration of maintenance treatment (mean + range) |
|---|---|---|---|---|
| Chemotherapy | 11 | 10 M<br>1 F | 10·1 years<br>(5–31) | 8·9 months<br>(3–14) |
| Immunotherapy (post-chemotherapy) | 20 | 7 M<br>13 F | 11·5 years<br>(4–21) | 28·2 months<br>(13–96) |
| Normals | 9 | 4 M<br>5 F | 30 years<br>(20–50) | — |

procedures by the absolute number of mononuclear cells/mm$^3$. Peripheral blood was collected in citrate from each subject and the mononuclear cells isolated and purified by a previously described Ficoll gradient procedure (Belpomme *et al.*, 1974). After at least three washings, these cells were used in the tests described below.

T lymphocytes were enumerated by the E rosette test using sheep red blood cells (SRBC). Rosettes were defined as lymphocytes surrounded by at least three SRBC. We performed this test in two ways. In the first, or direct test, rosettes resulting from the incubation of mononuclear cells and SRBC alone were counted (ERFC) (Belpomme *et al.*, 1974). In the second, or AB serum test, AB human serum, previously decomplemented and absorbed with SRBC, was added to the incubation mixture (EABRFC). We, as others, have previously shown that this latter procedure gives a higher number of rosettes than the former (Bentwich and Kunkel, 1973; Belpomme, personal communication). This phenomenon may be related to the detection of the total number of T cells by the sensitized test, while the direct test may define a sub-population of the group.

Enumeration of B lymphocytes was performed by determination of membrane immunoglobulin (mIg) using a direct immunofluorescent test with a polyvalent fluorescent isothiocyanate-conjugated sheep anti-human immunoglobulin serum. Details of this method have been previously described (Belpomme *et al.*, 1974).

Monocytes were enumerated by peroxidase staining as suggested by a recent WHO Workshop (Aiuti *et al.*, 1974). One thousand mononuclear cells were counted for peroxidase positivity on each slide.

After establishing the absolute number of each of the three foregoing groups (EABRFC, mIg$^+$ cells, peroxidase$^+$ cells) we calculated the number of so-called "null" cells by subtraction: "Null" cells = mononuclear cells − (EABRFC + mIg$^+$ cells + peroxidase$^+$ cells). Statistical analyses were performed by Student's *t* test.

Table II presents a summary of our results expressed in mean values, with ranges given in each instance. Since similar degrees of variation were seen in all groups, the use of mean values was considered justified.

The patients on chemotherapy had a significant reduction in number of mononuclear cells and of total number of EABRFC in comparison with both the immunotherapy and normal groups. No such difference was noted in either the total number of mIg$^+$ or peroxidase$^+$ cells. When "null" cells were calculated there was no difference from normals, but there were far fewer ($P = 0{\cdot}001$) in this group than in the patients receiving immunotherapy.

The immunotherapy group demonstrated no significant difference from the normal group except in the calculated "null cells". There was a much larger mean number of such cells in the immunotherapy group than in the normal ($P = 0{\cdot}01$).

In our study the percentages and total numbers of EABRFC, mIg$^+$ and peroxidase$^+$ cells in normal individuals were in the range previously published (Wybran

TABLE II.—*Summary of Results*

| | Mononuclear* cells | EABRFC* | mIg* | Peroxidase* | " Null " * | " Null " † |
|---|---|---|---|---|---|---|
| Chemotherapy | (667–2500) 1470 | (44–1180) 696 | (67–1400) 455 | (0–728) 344 | (0–460) 104 | (7%) |
| Immunotherapy | (1200–5360) 2714 | (456–2081) 1173 | (125–1126) 467 | (0–807) 198 | (174–1824) 883 | (33%) |
| Normal | (1100–3900) 2356 | (517–1863) 1299 | (110–720) 381 | (60–936) 396 | (0–1209) 304 | (12%) |
| Comparisons | | | | | | |
| Chemotherapy/immunotherapy | $P \leq 0 \cdot 001$ | $P = 0 \cdot 01$ | N.S. | N.S. | $P \leq 0 \cdot 001$ | |
| Normal/chemotherapy | $P = 0 \cdot 02$ | $P = 0 \cdot 01$ | N.S. | N.S. | N.S. | |
| Normal/immunotherapy | N.S. | N.S. | N.S. | N.S. | $P \leq 0 \cdot 01$ | |

* Number/mm$^3$ (range) and mean.

† As % mononuclear cells.

and Fudenberg, 1974). The calculated " null " cells were also in agreement with available data. Our control group was composed of subjects in an older age group than our patient groups because of the logistic difficulty of obtaining normal pediatric subjects. Several recent reports give conflicting results on the influence of age on the distribution of B and T cells. For example, one study (Weksler and Hutteroth, 1974) found no change in absolute number of peripheral lymphocytes or B and T cells with increasing age, while another (Carosella, Mochanko and Brown, 1974) demonstrated a decreased percentage of T cells occurring somewhere between 46 and 60 years of age. Since our control group had a mean age (30 years) considerably younger than this we believe that age difference does not represent a significant problem in analysis of our results.

A striking finding of our study is the significant elevation in the absolute number and percentage of " null " cells in the immunotherapy group as compared with both the chemotherapy and normal groups. Although the nature of these cells is still unknown, several hypotheses concerning their increase in this situation can be entertained.

(1) These cells may represent abnormal elements persisting even during apparently complete remission of acute lymphocytic leukaemia. The continued perfect clinical and cytological condition of these patients does not favour this hypothesis.

(2) They may be stem cells circulating in the peripheral blood. Although it has been demonstrated that BCG can increase haematopoietic stem cells in mouse bone marrow (Pouillart, personal communication) human data are lacking.

(3) They may be " K " cells. It has been shown that there is increased " K " cell activity in patients on BCG therapy for acute lymphocytic leukaemia in comparison to patients receiving no treatment (MacLennan, 1975). This hypothesis seems plausible since it has recently been suggested that " K " cells may be " null " cells (Greenberg *et al.*, 1973).

(4) They may represent T or B lymphocytes or monocytes which have lost any detectable markers, a change possibly induced by immunotherapy.

(5) A final possibility is that there may not be a true increase in these cells, but rather a redistribution between the peripheral blood and the various reticulo-endothelial organs.

Further studies are in progress in our laboratory to confirm these preliminary results and to elucidate the nature of these intriguing cells.

We gratefully acknowledge the technical cooperation of Daniele Grandjon and the statistical assistance of G. Hauss.

### REFERENCES

AIUTI, F. *et al.* (1974) Identification, Enumeration and Isolation of B and T Lymphocytes from Human Peripheral Blood. *Scand. J. Immun.*, **3**, 521.

BELPOMME, D. DANTCHEV, D. DU RUSQUEC, E., GRANDJON, D., HUCHET, R., POUILLART, P., SCHWARZENBERG, L., AMIEL, J. L. & MATHÉ, G. (1974) T and B Lymphocyte Markers on the Neoplastic Cells of 20 Patients with Acute and 10 Patients with Chronic Lymphoid Leukemia. *Biomedicine*, **20**, 109.

BENTWICH, Z. & KUNKEL, H. G. (1973) Specific Properties of Human B and T Lymphocytes and Alterations in Disease. *Transplant Rev.*, **16**, 29.

CAROSELLA, E. D., MOCHANKO, K. & BROWN, M. (1974) Rosette-forming T Cells in Human Peripheral Blood at Different Ages. *Cell. Immun.*, **12**, 323.

GREENBERG, A. H., HUDSEN, L., SHEN, L. & ROITT I. M. (1973) Antibody-dependent Cell-mediated Cytotoxicity Due to a " Null " Lymphoid Cell. *Nature* (*New Biol.*), **242**, 111.

GUTTERMAN, J. U., MAVLIGIT, G. M., REED, R. C. & HERSH, E. M. (1974) Immunochemotherapy of Human Cancer. *Seminars in Oncology*, **1**, 409.

HALPERN, B. N., BIOZZI, G., STIFFEL, C. & MOUTON, D. (1959) Effet de la Stimulation du Système Reticulo-endothelial par L'Inoculation du Bacille de Calmette-Guérin Sur le Développement L'Epithelioma Atypique T-8 de Guérin Chez le Rat. *C. r. Soc. Biol.* **153**, 919.

MACLENNAN, I. C. M. (1975) Immunosuppression and Immunostimulation in Acute Leukemia. *Proc. R. Soc. Med.*, **68**, 216.

MATHÉ, G., AMIEL, J. L., SCHWARZENBERG, L., SCHNEIDER, M., CATTAN, A., SCHLUMBERGER, J. R., HAYAT, M. & DE VASSAL, F. (1969) Active Immunotherapy for Acute Lymphoblastic Leukemia. *Lancet*, i, 697.

MATHÉ, G., KAMEL, M., DEZFULIAN, M., HALLE-PANNENKO, O. & BOURUT, C. (1973) An Experimental Screening for " Systemic Adjuvants of Immunity " Applicable in Cancer Immunotherapy. *Cancer Res.*, **33**, 1987.

NATHANSON, L. (1974) Use of BCG in the Treatment of Human Neoplasms: A Review. *Seminars in Oncology*, **1**, 337.

OLD, L. J., BENACERRAF, B., CLARKE, D. A., CARSWELL, E. A. & STOCKERT, E. (1961) The Role of the Reticuloendothelial System in the Host Reaction to Neoplasia. *Cancer Res.*, **21**, 1281.

WEKSLER, M. E. & HUTTEROTH, T. (1974) Impaired Lymphocyte Function in Aged Humans. *J. clin. Invest.*, **53**, 99.

WYBRAN, J. & FUDENBERG, H. (1974) How Clinically Useful is T and B Cell Quantitation? *Ann. intern. Med.*, **80**, 765.